# *Nursing Reports*: Annual Report Card 2021

**DOI:** 10.3390/nursrep12020038

**Published:** 2022-05-31

**Authors:** Richard Gray

**Affiliations:** School of Nursing and Midwifery, La Trobe University, Melbourne 3086, Australia; r.gray@latrobe.edu.au

As Editor-in-Chief of *Nursing Reports,* the focus of my work is to ensure submitted manuscripts are handled in a timely manner and that authors are provided with helpful and constructive feedback. I also want to be engaged and transparent with authors, reviewers, editorial board members and editors regarding the performance of the journal and how we might work to improve what we do and how we work. One of the ways I promote transparency is by publishing an annual report about how we are doing as a journal against a range of metrics. I published my first annual report for 2020 which covered the first six months of MDPI taking over as the publisher of *Nursing Reports* [1]. My second annual report covers all papers published in 2021. The report will essentially follow the same structure as last year. One additional area of focus is the gender balance of different groups of colleagues—associate editors and editorial board members—contributing to the journal.

*Goals from 2021*

In my previous report, we set some goals for *Nursing Reports* for the coming year [1]. In summary, we aimed to publish 60 papers and six editorials, grow the editorial board by appointing 25 new colleagues, maintain timely review and publication times, encourage more letters to the editor, implement pre-registration awards, publish two Special Issues, introduce registered reports, and grow the number of followers the journal has on social media. In this report, I will address our performance against these goals and other work we have undertaken.

## 1. Report Card

### 1.1. The Committee on Publication Ethics (COPE)

An important starting point is to restate that MDPI—the publisher of *Nursing Reports*—is a member of COPE and that all members of the editorial team follow the COPE code of conduct and best practice guidelines for journal editors. The COPE code is a set of minimum standards that editors are expected to adhere to. More information about the COPE code can be accessed via this link: https://publicationethics.org/files/Code_of_conduct_for_journal_editors_Mar11.pdf (accessed on 7 March 2011). Please make me or any of my editorial colleagues aware if you think our editorial practices fall below these standards.

### 1.2. Associate Editors

Currently, *Nursing Reports* has three Associate Editors, which is down one from the four we had in 2020. All are women. Two work in North America and one in Australia.

### 1.3. Editorial Board Members

The Editorial Board of *Nursing Reports* comprises 31 colleagues from nine countries. The number of editorial board members has decreased one since the last report. Board members are mainly from Europe (*n* = 17), Australia (*n* = 6) and North America (*n* = 5). Growing the size of the editorial board was a goal for 2021 which we did not achieve. Ten of our editorial board members are highly cited researchers.

### 1.4. Peer Reviewers

In total 227 colleagues have reviewed papers for *Nursing Reports*, for which I am personally extremely grateful. Most reviewers were from Europe (*n* = 132), North America (*n* = 48) and Asia (*n* = 20). No journal can function without peer reviewers who freely give their time to provide a rigorous critique of their colleagues' work; on behalf of MDPI and the editorial team, we thank you.

### 1.5. Documents Submitted

In total 186 papers were submitted to *Nursing Reports* in 2021. Most were reporting primary research (*n* = 154) or reviews of the literature (*n* = 28).

### 1.6. Documents Published

*Nursing Reports* published 90 papers—an acceptance rate of 60%—of which 68 papers were observational studies (cohort studies, surveys) and 9 were qualitative studies. In total, 13 (14.4%) papers were reviews of the existing literature (*n* = 3 systematic, *n* = 3 scoping, *n* = 7 other). Additionally, we published two editorials. We exceed our target in terms of the number of papers but not the number of editorials we were aiming for. We received fewer letters to the editor than we were hoping for.

### 1.7. Peer-Review Process

All papers—except for letters to the editor and editorials—submitted to *Nursing Reports* are reviewed by at least two reviewers. Reviewers are selected by the editorial team by matching keywords in the paper with those in the reviewer profile. The modal (average) number of reviews for each paper received was 2.7. The mean time from submission to first decision was 23.5 days (range 8–64 days), which was broadly similar to those we reported last year. As intended, we have maintained our handling times for submitted manuscripts.

### 1.8. From Acceptance to Publication

Once authors have received a formal acceptance notification from MDPI the average time to online publication is 5 days.

### 1.9. Appeals against Editorials Decisions

*Nursing Reports* has—as is required by the COPE code—a clear and transparent appeals process. If an author feels that their work has been unfairly rejected by an editor, they should feel empowered to appeal knowing that the decision will be reviewed by another editor who was not involved with the initial decision. It could be interpreted positively that *Nursing Reports* received no appeals against any of the editorial decisions that we made in 2021. However, I might express some concern that authors have not felt willing to challenge decisions they consider to be wrong.

### 1.10. Data Availability Statements

There is an increasing recognition that authors should make their data freely available for other researchers to check and reuse (for example, by combining datasets from similar studies) and MDPI is committed to encouraging and supporting authors to share and archive their research data publicly accessible repositories. Of the 90 papers published in 2021, 4 authors made their data available in a publicly accessible repository and 31 authors indicated that data were available on request.

### 1.11. Pre-Registration of Studies

Much has been written about the importance of pre-registering research before fieldwork starts or, in the case of systematic reviews, authors undertake their initial searches [2]. Pre-registration is important for multiple reasons but primarily because it reduces the risk of selective outcome bias [3,4]. I have written extensively on the importance of pre-registration and on how nursing as a discipline has been slow to embrace this agenda, see for example [5]. None of the primary research studies we published in 2021 were pre-registered.

#### Publishing Protocols and Registered Reports

One way in which journals can support the pre-registration agenda is through the publication of research protocols. Protocols are detailed descriptions of the study justification and methodology that a research group intends to follow. By publishing a protocol researchers make open and transparent the work they are doing. Since 2021, we have published seven protocols (*n* = 1 experimental study, *n* = 1 observational research, *n* = 5 reviews).

Another way in which *Nursing Reports* is supporting pre-registration is through the introduction of registered reports, one of our goals for the year. A registered report is a two-step publication process; part one is the protocol for the study that is published before the research starts. At this time the journal editor and author make an “in principle” agreement to publish the results of the study no matter the outcome. Registered reports help reduce the risk of publication bias, particularly where editors may be disinclined to publish “negative” findings from studies [6]. It is a little disappointing that, to date, we have not published any registered reports. We encourage authors to consider this approach to publishing that other disciplines have seemingly embraced more enthusiastically than nursing [6].

### 1.12. Reads, Downloads and Citations

The papers that were published in *Nursing Reports* were read and downloaded a total of 116,243 and 91,112 times, respectively, in 2021; a 2287% and 2393% increase over 2020 (based on data from the journal’s website). In total papers published in *Nursing Reports* received 84 citations (in WoS) in 2021.

### 1.13. Special Issues

Special Issues serve as a useful mechanism for bringing together researchers and scholars to focus on a particular topic. Currently, we have five active *Nursing Reports* Special Issues in progress (https://www.mdpi.com/journal/nursrep/special_issues, accessed on 25 May 2022): New Advances in Nursing Care, Evidence-Base Practice and Personalized Care, Burnout and Nursing Care, Nursing and COVID-19, Quality of Life in Cancer Patients. 

### 1.14. Social Media

*Nursing Reports* has established a Twitter account (@NursRep_MDPI) which we launched in February 2021. To date, we have 112 followers and have published 129 tweets. I would encourage colleagues to follow us on Twitter and retweet our content that interests or engages you.

### 1.15. Indexing

*Nursing Reports* is listed on Clarivate Analytics Emerging Sources Citation Index (ESCI). ESCI was launched in 2015 to ensure visibility of research in the Web of Science Core collection and is a stepping stone to inclusion in the Science Citation Index Expanded (SCIE) if journals consistently demonstrate the highest standard of editorial and publishing best practices. Over the course of the rest of 2022, we will continue to work towards the twin goals of being listed in SCOPUS and the SCIE which is important in affirming the reputation of the journal across the broader nursing science community.

### 1.16. Editorial Position on Empty Reviews

A small—but in my view important—achievement in 2021 was for *Nursing Reports* to take a positive position on publishing empty reviews. An empty review is one where no studies meet the pre-defined inclusion criteria. If you would like to read more about empty reviews, I would direct readers to the work of Yaffe et al. [7]. Empty reviews can make an important contribution by identifying gaps in knowledge that can be addressed by future research. Editors—in my experience—can be reluctant to publish empty reviews (and indeed reviews where only a few papers are included), not because of the quality of the work, but rather because of a misplaced idea that reviews need a certain number of papers to draw meaningful conclusions.

### 1.17. Best Paper Awards

The winner will not be announced until March 2023, but *Nursing Reports* has launched a best paper award. In fact, two awards—best review and best research—for papers published in 2021. Winners of the awards will receive 500 CHF and a voucher to publish a paper in the Journal. More importantly, winners will receive a certificate. If you would like more information about the awards, please see the following link (https://www.mdpi.com/journal/nursrep/awards, accessed on 25 May 2022). Rest assured we will have journal awards for the best papers published in 2022.

### 1.18. Inclusive Language

A final, but again important, achievement (although this technically happened in 2022) is that we have published guidance encouraging authors that contribute to *Nursing Reports* to be careful and considered in the language they use. Simply put, words matter. You can read more about the importance of inclusive language in our revised instructions for authors (https://www.mdpi.com/journal/nursrep/instructions, acceded on 25 May 2022).

## 2. Challenges and Opportunities for 2022

For the year ahead—recognizing that we are already well into 2022—these are what I see as the challenges and opportunities that—with my editorial colleagues—I want to address.

### 2.1. Working Closely with the Editors and the Editorial Board

The editorial board will meet three times in 2022 to review the performance of the journal and how we might work together to better address the challenges and concerns of authors. We will also look to expand the membership of the editorial board, with a particular focus on colleagues from underrepresented regions. If you are interested in joining the editorial board, please contact me or my MDPI colleagues.

### 2.2. Patient and Public Involvement and Engagement in Research

There is a consensus view that patient and public involvement and engagement (PPIE) in research improves the quality, relevance, and impact of the work. In many countries funding bodies require that researchers demonstrate how patients and the public have been authentically and meaningfully involved in the design of the research. There is little evidence that nurses are working with patients and the public in the design of their research [8]. I would like to encourage nurse researchers to actively engage stakeholders in their research and explain in the papers from that work how they did this. In the author guidelines for *Nursing Reports*, we do encourage authors to describe how patients and the public were involved in the submitted research. Working with the editorial board we will develop a plan about how we support meaningful engagement of patients and the public in research.

### 2.3. Adherence to Reporting Guidelines

Guidelines such as CONSORT [9] are intended to enhance the quality and precision of research reporting [10]. Few authors when submitting papers to *Nursing Reports* indicate that they have followed the relevant guideline. In 2022, we will make it much clearer to authors that they need to ensure that their reporting is fully compliant with the relevant guideline and ensure that this has been done prior to manuscripts being sent for peer review.

### 2.4. New Special Issues

In 2022, we would like to instigate at least another six Special Issues; please contact me if you would like to discuss this further.

### 2.5. More Editorials

It would be good to see more editorials in *Nursing Reports*. We will set a goal of publishing at least six editorials in 2022 and would encourage authors with a topic or issue that would like to write about, again, to contact me. I will also actively encourage members of the editorial board to contribute editorials to the journal.

## 2.6. Acknowledging the Contribution of Peer Reviewers

We will publish a list annually (likely in January 2023) acknowledging and thanking peer reviewers that have contributed to *Nursing Reports* in 2022. We will also award a prize at the end of the year to the reviewer that has reviewed most papers for the journal.

### 2.7. Encouraging Letters to the Editor

Post-publication scrutiny is a vital part of the scientific method [11]. Unlike other clinical disciplines, nurses are seemingly reluctant to put pen to paper and offer critiques of published research. I made this observation in last year’s report, and there is little evidence of improvement. I would encourage readers who identify issues with papers to consider writing and submitting a letter to the editor. As a discipline, we must become more engaged in critiquing the work that we use to underpin our clinical practice.

### 2.8. Ensuring Data Are Freely Available

I am not convinced that nurse researchers have grappled with the need to ensure that the data that underpins the research that they publish is freely available for other researchers to check and reuse. I intend to write an editorial setting out the journal's position on data availability and how nurses—when designing their research—should consider carefully how the data from their study will be curated at the end of the project.

### 2.9. Editorial Position on Pilot and Feasibility Studies

Several other nursing journals have an editorial position not to publish pilot or feasibility studies. This type of research is important in informing full studies. Researchers should not, for example, embark on a full randomized controlled trial without first establishing that it is feasible to recruit the required number of participants. By not publishing pilot or feasibility studies, in my view, journals are sending a message to authors that this work is not important; in fact, the opposite is true. Well-conducted preliminary research is important in ensuring that time, effort, and resources are not wasted and that participants are not put at unnecessary risk. *Nursing Reports* will adopt a positive editorial position encouraging authors to submit their pilot and feasibility work.

## 3. Final Comments

By publishing an annual report as Editor-in-Chief of *Nursing Reports,* my MDPI colleagues and I are endeavoring to be as open and transparent with our editors, reviewers, authors, and readers as we can be. If there is information that you think is helpful for us to include in the next report, please do get in touch.

The editorial philosophy of *Nursing Reports* is to be author-focused. We aim to ensure that researchers and scholars that submit their work to the journal receive positive, constructive, and timely reviews of their work. Because *Nursing Reports* is Open Access, papers are not hidden behind a paywall and can be freely accessed by nurses in practice, and the patients and families they work with.

## Data Availability

Data sharing not applicable—no new data generated.
